# Scapular Winging following Sports-Related Injury in a Rugby Player

**DOI:** 10.1155/2021/4511538

**Published:** 2021-10-26

**Authors:** Shinya Ishizuka, Akinori Kobayakawa, Hideki Hiraiwa, Hiroki Oba, Takefumi Sakaguchi, Masaru Idota, Takahiro Haga, Takafumi Mizuno, Itaru Kawashima, Kanae Kuriyama, Shiro Imagama

**Affiliations:** Department of Orthopedic Surgery, Nagoya University Graduate School of Medicine, Nagoya, Japan

## Abstract

The most common cause of medial scapular winging is long thoracic nerve palsy (LTN) and subsequent serratus anterior muscle dysfunction. A 16-year-old right-handed male high-school rugby player developed severe right-sided neck and shoulder pain after tackling an opponent while playing rugby. Six weeks after initial injury, the patient observed shoulder muscle weakness when performing his daily activities. On physical examination, limited active elevation of the right shoulder in the scapular plane and scapular winging was observed. Magnetic resonance imaging revealed atrophy of both the SA and subclavius muscles on the right side, and we initially suspected an LTN injury sustained. However, while detailing his history, the patient explained that he also had noted difficulty sucking high viscosity drinks such as shakes and smoothies since childhood. In addition, physical examination showed weakness of the orbicularis oculi muscle. Considering the facial muscle weakness, facioscapulohumeral dystrophy (FSHD) was also suspected, and genetic testing showed chromosome 4q35 deletion with restriction fragments 17 kb and 3 tandem repeated DNA confirming the diagnosis of FSHD. Clinicians should be aware that FSHD could be one of the differential diagnoses of scapular winging after sports injury, and surgeons should rule out the diagnosis of FSHD before performing any surgical treatment for SA palsy.

## 1. Introduction

The most common cause of scapular winging is long thoracic nerve (LTN) palsy caused by trauma or overuse during sports activity and subsequent serratus anterior (SA) muscle dysfunction [1, 2]. Facioscapulohumeral dystrophy (FSHD), the third most common type of dystrophy, causes asymmetric atrophy of the muscles of the face, upper arm, and shoulder girdle, leading to scapular winging. To the best of our knowledge, there are no case reports of FSHD diagnosed based on scapular winging after a sports-related injury. Herein, we report about an otherwise healthy 16-year-old high school rugby player who presented with complaints of pain and decreased range of motion (ROM) of the right shoulder, as well as unusual scapular winging on the right side for 2 months after a tackle during a rugby game. Clinicians should consider another underlying disorder if the presumed cause does not explain the symptoms sufficiently.

## 2. Case Presentation

**Figure 1:**
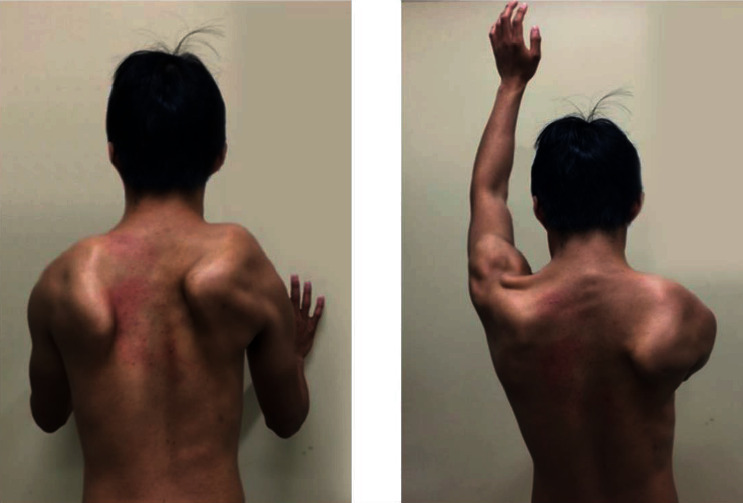
Bilateral scapular winging (right > >left) during (a) wall push test and (b) active shoulder flexion. The patient is unable to elevate his right arm to a horizontal level.

A 16-year-old right-handed male high-school rugby player developed severe right-sided neck and shoulder pain after tackling an opponent while playing rugby. After resting for a few days, the shoulder pain improved, and he began to play rugby again for his high school rugby league. After the initial injury, however, he occasionally experienced similar pain while playing rugby, which resolved after a few days of rest. The primary orthopedist suspected shoulder muscle strain for which the patient was referred to a clinic where he started physical therapy of the shoulder. He underwent 4 weeks of physical therapy, which involved simple strengthening exercises, to restore his ROM and reduce inflammation; not only pain did not improve, but he also now had persistent shoulder pain—initially, he experienced pain only when moving the shoulder. Six weeks after initial injury, the patient observed shoulder muscle weakness when performing his daily activities. When he was referred to our clinic 2 months after the initial injury, his chief complaint was limited shoulder elevation, not pain. He had no known family history and past history. On physical examination, active elevation of the right shoulder in the scapular plane was limited to 50°, whereas the ROM of his left shoulder was normal. Scapular winging produced by the wall push test and active shoulder elevation was observed bilaterally (right > >left) (Figure 1).

**Figure 2:**
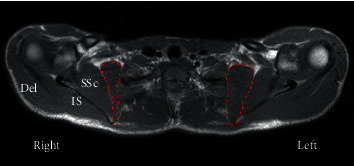
*T*1-weighted magnetic resonance image showing slight atrophy of the serratus anterior muscle (dotted line) on the right side compared with left side in the axial plane. SSc: subscapularis muscle; IS: infraspinatus muscle; Del: Deltoid muscle.

The patient's rotator cuff tests and impingement signs were negative in his right shoulder. Manual muscle testing did not show any weakness of the biceps, triceps, or grip strength. Plain radiographs had no abnormal findings. However, magnetic resonance imaging (MRI) revealed atrophy of both the SA and subclavius muscles on the right side, but no significant pathology was observed in the shoulder joint or cervical spine (Figure 2).

Blood test results showed slight elevations of creatine kinase (CK; 870 U/L), alkaline phosphatase (526 U/L), lactate dehydrogenase (LDH; 370 U/L), and aldolase (10.9 U/L). While detailing his history, the patient explained that he also had noted difficulty sucking high viscosity drinks such as shakes and smoothies since childhood. In addition, physical examination showed weakness of the orbicularis oculi muscle. Initially, we suspected neuralgic amyotrophy or an LTN injury sustained when playing rugby. However, considering the facial muscle weakness, FSHD was also suspected, and genetic testing was ordered to confirm the diagnosis before performing invasive examinations such as electromyography (EMG). Although he had no family history of muscular dystrophy, genetic testing performed at an outside laboratory showed chromosome 4q35 deletion with restriction fragments 17 kb and 3 tandem repeated DNA (D4Z4) confirming the diagnosis of FSHD. Since the diagnosis of FSHD, he is being followed up periodically to check for any progress of the muscle weakness or development of FSHD-related complications. The patient was informed that data from the case would be submitted for publication and gave their consent.

## 3. Discussion

In this report, we describe an unusual case of scapular winging in a young patient after he sustained an injury while playing rugby, which was finally diagnosed as FSHD.

Scapular winging causes discomfort, decreased shoulder strength, and ROM [2]. Scapular winging is divided into two types (medial > lateral) [3], and our patient had the medial type. Previous studies reported that the main causes for medial scapular winging are SA dysfunction following LTN palsy [1, 3] or traumatic SA muscular detachment [4–6]. Other studies have reported that trauma or overuse related to sports activity resulted in 40-90% cases of LTN palsy [7, 8]. In addition, most cases of LTN injury involve the dominant side, similar to our patient [9]. Thus, SA palsy caused by an LTN injury was initially suspected in this case.

FSHD is often cited as the third most common muscular dystrophy characterized by slow progressive asymmetric atrophy of the facial, shoulder, upper arm, and leg muscles [10, 11]. The primary symptoms are scapular protrusion, shoulder pain owing to scapulothoracic instability, or decrease of shoulder strength and ROM. Patients with FSHD usually notice a gradual progression of these chronic symptoms without any inciting events. The slow progression of the condition sometimes makes it difficult for patients to be aware of their own discomfort. Patients with FSHD primarily consult neurologists, pediatricians, and often orthopedists. Hence, FSHD should be widely understood by orthopedists and orthopedic surgeons.

Unfortunately, the diagnosis is often delayed despite symptoms of chronic pain and disability. To avoid a misdiagnosis of FSHD, specific diagnostic questions that focus on facial weakness could be useful, such as “Are you good at whistling?”, “Are you good at smiling?”, “Do you feel difficult while drinking shakes?”, and “Do you sleep with eyes open?” These symptoms caused by the weakness of facial muscles tend to be observed from the early phase of disease. In our case, since the patient experienced some difficulty in drinking shakes and smoothies, he had been avoiding it from childhood. Although an LTN injury can be confirmed using EMG following a medical interview and general examination, we performed genetic testing before the EMG, because it is invasive and painful for patients. FSHD is confirmed by genetic testing which shows the deletion of tandemly repeated DNA (D4Z4 repeats) in chromosome 4q35. In some cases, slight elevations of CK, LDH, or aldolase are observed in blood testing, though these variables are not helpful for the diagnosis [12].

Unfortunately, there is no cure or medical treatment available for FSHD. Most cases of LTN injury recover with conservative management, and recovery can sometimes takes several months to a few years [1, 13–15]. If FSHD had been misdiagnosed and treated conservatively as an LTN injury, it would have resulted in delayed diagnosis or gone undiagnosed. Surgical treatment has some disadvantages as well, for patients. There are several surgical options for SA palsy following an LTN injury, such as LTN release [16], scapulothoracic arthrodesis, and pectoralis major transfer [2, 17]. LTN release has no effect on muscular dystrophy and thus should not be performed for FSDH. The indications of scapulothoracic arthrodesis include both LTN palsy and FSHD [18]. However, pectoralis major transfer for dynamic stabilization of scapula should be avoided in patients with FSHD, because pectoralis major is one of the affected muscles in FSHD.

In our patient diagnosed with FHSD, the cause of neck and shoulder pain following a traumatic event is unclear. The most disabling dysfunction of FSHD is the lack of fixation of the scapula against the thoracic cage during shoulder flexion and abduction [9], and in this situation, the other muscles stabilizing the scapula, including the trapezius, rhomboids, and levator scapulae, would only partially compensate for the SA function. We speculate that excessive force to these muscles while playing rugby caused muscle strain and pain. In fact, the painful sites corresponded to the locations of these muscles.

To the best of our knowledge, this is the first case report of FSHD with unusual scapular winging and limited shoulder ROM after a traumatic event. Physiotherapists, orthopedists, and orthopedic surgeons should be aware that FSHD could be one of the differential diagnoses of scapular winging after sports injury, and surgeons should rule out the diagnosis of FSHD before performing any surgical treatment for SA palsy.

## Data Availability

The data that support the findings of this study are available from the corresponding author, [author initials], upon reasonable request.
